# A Spectrum of Hematological Disorders in Children with Pancytopenia Based on Bone Marrow Examination in a Tertiary Care Hospital

**DOI:** 10.7759/cureus.5124

**Published:** 2019-07-11

**Authors:** Raana Zeeshan, Bilal Irshad, Muhammad A Aslam, Muhammad T Khan, Hamza Waqar Bhatti, Noman A Chaudhary

**Affiliations:** 1 Pathology, Benazir Bhutto Hospital, Rawalpindi, PAK; 2 Pathology, Rawalpindi Medical University, Rawalpindi, PAK; 3 Internal Medicine, Rawalpindi Medical University, Rawalpindi, PAK; 4 Surgery, Rawalpindi Medical University, Rawalpindi, PAK

**Keywords:** pancytopenia, bone marrow examination, hematological disorders

## Abstract

Introduction

Pancytopenia is a common presentation in the pediatric population. It is a manifestation of various diseases, and its etiology can be explained on the basis of bone marrow examination. The study aims to determine the etiological factors leading to pancytopenia via bone marrow examination in pediatric patients presenting in our hospital.

Materials and methods

This retrospective cross-sectional study was conducted in the Department of Pathology at a public sector tertiary care hospital. Data were recorded by convenience sampling from the patients’ database from January 2015 to April 2018. Patients aged 2 months to 15 years who had pancytopenia on peripheral blood smear and were admitted for bone marrow examination were included in the study. Patients who were beyond these age limits, diagnosed cases of aplastic anemia and leukemia, and those with a recent history of blood transfusion were excluded from the study. The analysis was done via the Statistical Package for Social Sciences (SPSS) v.23.0 (IBM SPSS Statistics, Armonk, NY, USA), and descriptive statistics were applied.

Results

Of 115 cases, 58 (50.4%) were males and 57 (49.6%) were females. Megaloblastic anemia was present in 32 (27.8%) patients, and it was the most common cause of pancytopenia. Non-malignant disorders were seen in 95 cases (82.6%) and malignant disorders were seen in 20 (17.4%) cases.

Conclusion

Megaloblastic anemia is the most common cause, and acute lymphoblastic leukemia is the most common malignant cause of pancytopenia in children. There was no significant gender predilection among causes of pancytopenia.

## Introduction

Pancytopenia is a condition in which there is a decrease of all the three cellular elements of blood, namely erythrocytes, leukocytes, and platelets [1-5]. It is quite a common finding among children. According to regional studies, it makes up around 2.90% to 3.57% of the burden of presentations in the pediatric population [3, 5]. Some of the presenting symptoms include, but are not limited to, pallor, fever, weight loss, dyspnea, bleeding, bruising, visceromegaly, and increased risk of infections [2, 4, 6].

Pancytopenia is not a disease in itself but a laboratory finding of the constitution of diseases [2, 3, 7]. It can occur due to a variety of disorders which can be as simple as nutritional deficiencies to serious malignant disorders [5, 6]. Megaloblastic anemia and acute lymphoblastic leukemia are among the most common presentations of pancytopenia [2-4, 8].

Pancytopenia results in a hypoplastic bone marrow [9]. It is diagnosed on the basis of the complete blood count test [10]. Pancytopenia is labeled when the hemoglobin (Hb) is less than 10 gm%, absolute neutrophil count (ANC) is less than 1.5*10^9^/L, and platelet count is less than 100*10^9^/L [9].

But, in order to find out the etiological factor causing pancytopenia, bone marrow examination is done [3, 4]. It consists of taking a sample from the bone marrow of a patient and then studying its components under a microscope [10]. Being an interventional procedure, it is relatively safe with little or no risk of bleeding [3, 6, 7].

The aim of this study was to determine the spectrum of pancytopenia with its frequency and etiology on the basis of bone marrow examination in children presenting to our hospitals. This has been an area of great interest in South-East Asia, and many articles on a similar topic have been published recently. Studying about the etiology of pancytopenia will equip us with better knowledge on how to tackle this pathology.

## Materials and methods

The study was a retrospective descriptive cross-sectional study. It was conducted in the Department of Pathology at a public sector tertiary care hospital. The ethical approval was sought from the Institutional Research Forum of Rawalpindi Medical University. The data were collected by convenience sampling from the pathology department of the hospital, in accordance with the Declaration of Helsinki. The patients' identities were kept anonymous and only the serial number, gender, and etiology for pancytopenia were noted. Patients’ database was explored for patients with pancytopenia during the period of January 2015 to April 2018. Pancytopenia was defined as Hb < 10 gm%, ANC < 1.5*10^9^/L, and platelet count < 100*10^9^/L. Patients aged 2 months to 15 years who had pancytopenia on a peripheral blood smear and were admitted for bone marrow examination were included in the study. Patients who were beyond these age limits, diagnosed cases of aplastic anemia and leukemia, and those with a recent history of blood transfusion were excluded from the study. The analysis was done using the Statistical Package for Social Sciences (SPSS) v. 23.0 (IBM Statistics, Armonk, NY, USA). Simple descriptive statistics were applied for finding out frequency distribution. Chi-square test was applied for finding out the association between gender and causes of pancytopenia, and p-value of less than 0.05 was considered significant. 

## Results

Of the 115 cases, 58 (50.4%) were males and 57 (49.6%) were females with a male to female ratio of 1.01:1. Megaloblastic anemia was the most common presentation of pancytopenia with 32 (27.8%) cases, followed by acute lymphoblastic leukemia with 16 (13.9%) cases. Non-malignant disorders were seen in 95 (82.6%) cases and malignant disorders were seen in 20 (17.4%) cases. Malignant disorders included acute lymphoblastic leukemia in 16 (13.9%) cases and acute myeloid leukemia in 4 (3.5%) cases. Non-malignant disorders included nutritional disorders in 43 (37.4%) cases and non-nutritional disorders in 52 (45.2%) cases. The further division is described in more detail in Table 1. Chi-square test was applied to show an insignificant association of gender with causes of pancytopenia (p>0.05).

**Table 1:** Spectrum of Hematological Disorders Found in Children with Pancytopenia (n=115)

Diseases		No. of cases	Percentage
Non-malignant disorders			
Nutritional disorders	Megaloblastic anemia	32	27.8
	Mixed deficiency anemia	6	5.2
	Iron deficiency anemia	6	5.2
Non-nutritional disorders	Infective destruction of cells	7	6.1
	Hypoplastic bone marrow	6	5.2
	Aplastic anemia	6	5.2
	Visceral leishmaniasis	6	5.2
	Hemolytic anemia	4	3.5
	Diluted marrow	4	3.5
	Chronic malaria	3	2.6
	Dyserythropoietic anemia	3	2.6
	Immune destruction of cells	3	2.6
	Myeloid maturation arrest	2	1.7
	Hemophagocytic syndrome	2	1.7
	Myelofibrosis	2	1.7
	Sideroblastic anemia	1	0.9
	Chronic granulomatous disease	1	0.9
	Erythroid and megakaryocytic depression	1	0.9
Total		95	82.6
Malignant disorders			
	Acute lymphoblastic leukemia	16	13.9
	Acute myeloid leukemia	4	3.5
Total		20	17.4

## Discussion

Pancytopenia is not an uncommon presentation in pediatric wards. A thorough history taking, physical examination, and the right laboratory investigations can lead to proper diagnosis and hence, to the relevant management of the etiology [6].

In the present study, males and females were in similar numbers with a male to female ratio of 1.01:1, but other studies reported different numbers. In the studies conducted by Makheja et al., Memon et al. in Karachi; Jan et al., Gul et al. in Peshawar; Tufail et al. in Faisalabad; Dubey et al. in India; and Basak et al. in Bangladesh, male to female ratios were 1.38:1, 1.6:1, 1.84:1, 1.8:1, 0.76:1, 0.88:1, and 1.7:1, respectively [2-8].

Non-malignant disorders were predominantly high in our study with 95/115 (82.6%) cases as compared to malignant disorders with 20/115 (17.4%) cases. Similar results were obtained in a study by Memon et al. with non-malignant disorders at 190/230 (82.59%) cases and malignant disorders at 40/230 (17.4%) cases [3].

Our study showed that megaloblastic anemia (27.8%) is the most common nutrition-related presentation of pancytopenia. This is consistent with studies done by Makheja et al. and Dubey et al. with megaloblastic anemia as the most common presentation at 41.9% and 47%, respectively [5, 6]. Various other studies have also reported megaloblastic anemia to be the most common non-malignant presentation of pancytopenia [3, 4]. These trends are because of the fact that nutritional anemias are quite common in the subcontinent due to the poor quality of food and dietary habits. The most common nutritional anemia in our region is vitamin B12 deficiency that also presents among the patients with pancytopenia [6, 9, 11].

Further results of our study showed that acute lymphoblastic leukemia (13.9%) is the most common malignant presentation of pancytopenia. The study by Memon et al. also reported similar results with acute lymphoblastic leukemia at 8.69% [3]. Jan et al. reported leukemia at 23.9%, whereas Tufail et al. confirmed similar findings with leukemia being at 28% [2, 9]. This increasing incidence may be related to urbanization, exposure to environmental chemicals, and radiation hazards.

Despite these important findings, the limitations of the study are also noted. This is a small center cross-sectional study done by convenience sampling; therefore, the results may not apply to other regions. Further studies are needed to complement these findings and study the trends in other parts of the world.

## Conclusions

Pancytopenia is related to nutritional deficiencies in our area. Megaloblastic anemia is the most common presentation of pancytopenia in children. The most common malignant presentation is acute lymphoblastic leukemia. There was no significant gender predilection among causes of pancytopenia.
